# The angular trajectory of the vestibular aqueduct: evaluation using photon-counting CT

**DOI:** 10.1186/s13244-026-02372-8

**Published:** 2026-08-25

**Authors:** Frederik Van den Kerkhof, Lukas Boomgaert, Lesley Cockmartin, Robert Hermans

**Affiliations:** 1 Department of Radiology, University Hospitals Leuven, Leuven, Belgium; 2 Department of Radiology, Sint-Trudo hospital, Sint-Truiden, Belgium; 3 Department of Imaging and Pathology, KU Leuven-University of Leuven, Leuven, Belgium

**Keywords:** Vestibular aqueduct, Temporal bone, Bony labyrinth, Ear (inner), Tomography (X-ray computed)

## Abstract

**Abstract:**

The angular trajectory of the vestibular aqueduct (ATVA) has been proposed as a radiographic marker for developmental endotypes of Menière’s disease. Current ATVA measurements rely on software-based proxies because the proximal vestibular aqueduct is often below the spatial resolution of conventional CT. Photon-counting CT (PCCT) may allow direct visualization of the proximal trajectory and enable patient-specific ATVA measurement. In this retrospective study, PCCT temporal bone scans from 64 patients (128 ears) were analyzed, and ATVA was measured directly by two independent readers, alongside additional VA morphometric parameters. The mean ATVA (106.9° ± 11.7°) was consistent with previously reported values, suggesting that PCCT can approximate established measurements without relying on geometric proxies. However, reproducibility was poor: interobserver agreement for ATVA was low (ICC = 0.24), and intra-observer agreement varied markedly between readers (ICC = 0.80 vs. 0.27). This variability can be related to subjective line placement, the curved anatomy of the VA, and the need to integrate information across multiple image slices. In contrast, midpoint VA diameter measurements, particularly in the Pöschl plane, demonstrated moderate to good reproducibility. Methodological standardization, such as the use of individualized oblique reconstructions or minimum intensity projections, may improve the reliability of ATVA measurements. Alternatively, other surrogate markers, such as the retrolabyrinthine bone thickness, may provide superior reproducibility while maintaining clinical relevance for endotype differentiation. Overall, despite enabling direct visualization, the current PCCT-based ATVA assessment is limited by poor reproducibility and requires methodological refinement for routine clinical implementation.

**Key Points:**

•**Question** Photon-counting CT can directly assess the angular trajectory of the vestibular aqueduct for Menière’s disease endotyping, avoiding software-based proxies used as conventional CT cannot resolve its proximal anatomy.

•**Findings** Photon-counting CT showed a mean ATVA of 106,9°, comparable to values previously reported in the literature, but with poor inter-reader agreement.

•**Critical relevance statement** Photon-counting CT enables direct patient-specific assessment of the angular trajectory of the vestibular aqueduct and may support clinically meaningful endotype categorization in Menière’s disease.

**Graphical Abstract:**

**Figure:**
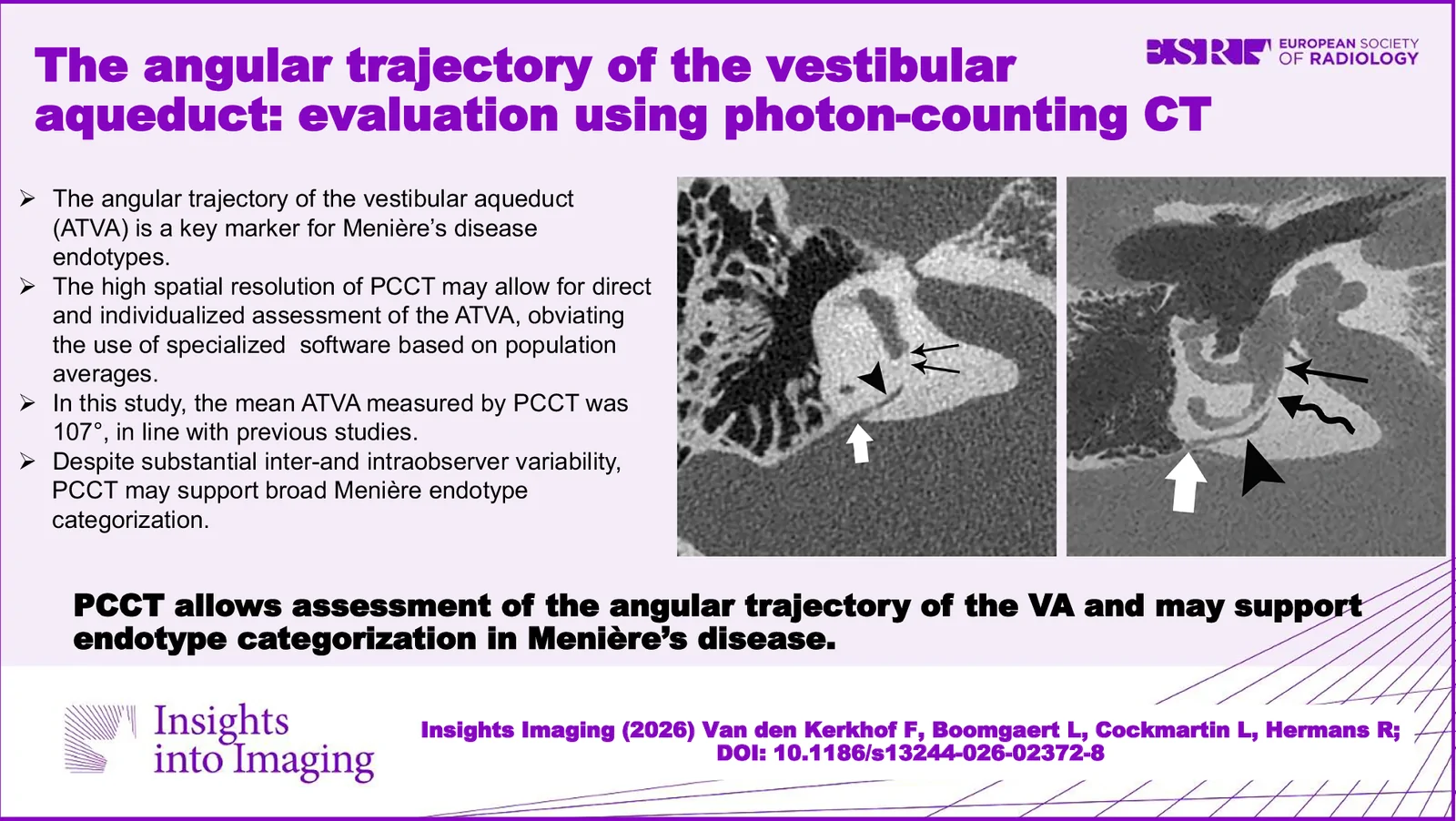

## Introduction

The angular trajectory of the vestibular aqueduct (ATVA) is a morphological parameter defined as the angle formed between the narrow proximal part and the descending part of the vestibular aqueduct (VA). During normal postnatal development, the ATVA typically undergoes a significant transition from a fetal or straight configuration to a mature curved adult form over the first decade of life [1].

The ATVA serves as a radiographic surrogate marker to distinguish between distinct endotypes of Menière’s disease (MD). Patients exhibiting a persistent fetal ATVA are classified as having the hypoplastic endotype (MD-hp), which is associated with underdevelopment of the endolymphatic sac. Clinically, these patients suffer from a higher prevalence of bilateral disease progression, a longer history of vertigo attacks, and an increased likelihood of progressing to profound hearing loss compared to those with the degenerative endotype (MD-dp). Typically, patients with MD-hp show an ATVA > 140°, while those with MD-dp typically will show smaller angles [2, 3].

Currently, the ATVA is measured using specialized software that accounts for the fact that the proximal part of the vestibular aqueduct is often below the spatial resolution of standard clinical CT and MRI. To overcome this, a predefined geometric shape is fit to the vestibule and lateral semicircular canal, and a line at a fixed angle of 14.3° is attached, representing the average entrance angle as determined by historical histological studies, to serve as a baseline proxy for the proximal trajectory [4]. Consequently, clinicians have been forced to rely on population-level averages rather than the unique anatomy of the individual patient. Furthermore, this approach requires exporting the imaging study to external software for processing to allow for this fitting and subsequent ATVA measurement, making the process cumbersome and hindering its applicability in routine clinical practice.

In this study, photon-counting computed tomography (PCCT) was used to evaluate the ATVA. The hypothesis is that the greater spatial resolution of PCCT compared to energy-integrating CT (EICT) would permit direct assessment of the proximal VA trajectory, thereby eliminating the need for surrogate proxies and possibly improving the precision of endotype classification for MD patients [5].

## Materials and methods

This retrospective, single-center study was approved by the local Research Ethics Committee of UZ/KU Leuven (reference: s69789); the requirement for informed consent was waived.

PCCT scans of the temporal bones (Naeotom Alpha; Siemens Healthineers, Forchheim, Germany) from 70 consecutive patients, acquired between November 2021 and June 2022, were reviewed. Six patients were excluded due to the presence of a cochlear implant (*n* = 2), motion artifacts (*n* = 3), or an incomplete examination (*n* = 1), resulting in 128 temporal bones available for analysis. The mean age of the included patients (*n* = 64) was 49 ± 21 years (range: 9–96 years). Scan acquisition parameters were as follows: 120 kV, 144 × 0.2 mm collimation, pitch 0.35, IQ level 75, and a 0.5 s rotation time.

The clinical indications for PCCT were conductive hearing loss or middle ear disease (*n* = 35), sensorineural hearing loss (*n* = 6), mixed hearing loss (*n* = 5), third-window pathology (*n* = 6), skull base tumor (*n* = 5), tinnitus (*n* = 4), skull base infection (*n* = 2). None of the patients exhibited inner ear abnormalities or evidence of labyrinthine capsule disease on CT images, as assessed by a senior head and neck radiologist with 35 years of experience. Additionally, none of the patients was suspected of having MD.

**Fig. 1:**
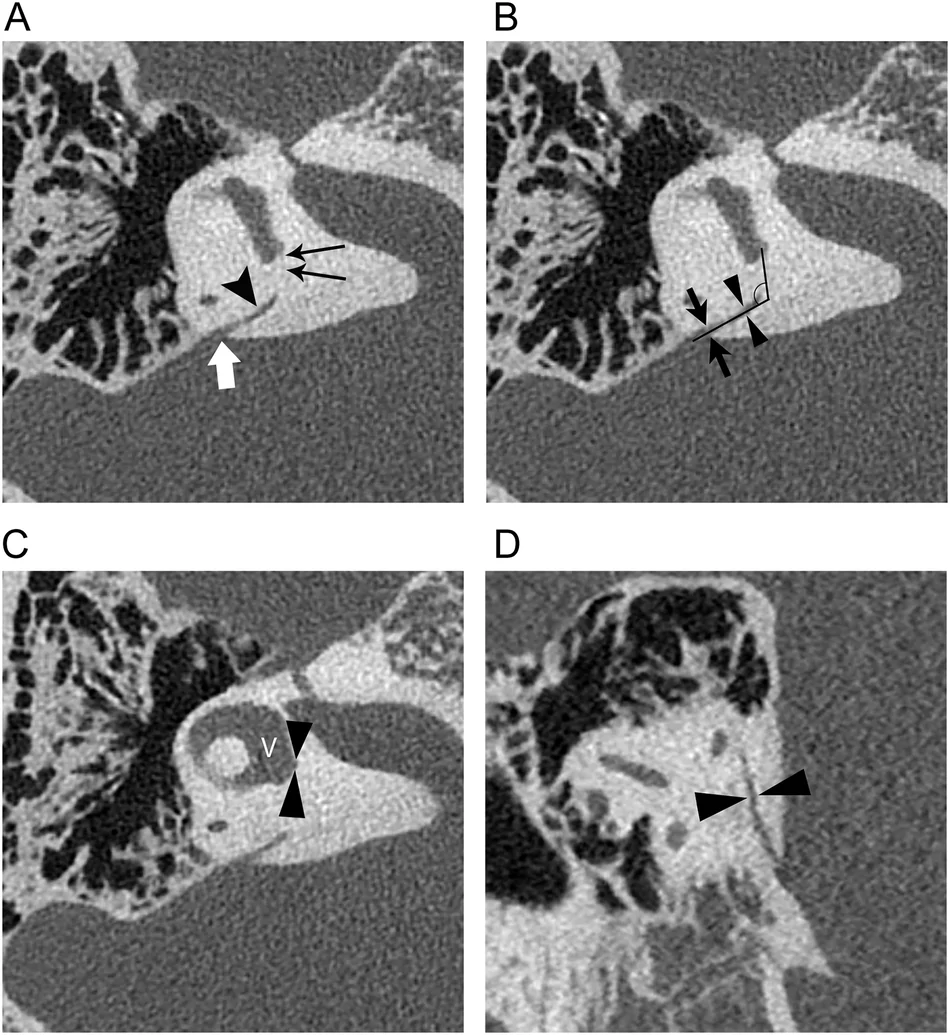
**A** Axial images along the plane of the lateral semicircular canal. Proximal VA segment is faintly visible (double arrow), distal VA segment (arrowhead), opercular segment (white arrow). **B** Same image as **A** showing lines drawn along the proximal and distal segments of the VA, and the angle between both lines (curved line). The midpoint width was measured between the arrowheads, and the opercular width between the arrows. **C** Image obtained along the same plane, more cranial section. The internal aperture of the VA is visible as a small dot on the side of the vestibule (V); its width was measured between the arrowheads. **D** Image along Pöschl’s plane showing VA in the same patient; the midpoint width was measured between the arrowheads

The native CT images were reformatted along the plane of the lateral semicircular canal using a PACS workstation (Enterprise Imaging; Agfa Healthcare, Mortsel, Belgium). This plane was selected to maintain consistency with previous studies [1, 2, 4, 6, 7]. On these axial images, the senior head and neck radiologist and a senior radiology resident independently measured the following parameters bilaterally for all patients: the width of the VA aperture (at the junction with the vestibule), the midpoint width of the VA, and the opercular width of the VA at its junction with the posterior fossa. The ATVA was defined as the angle between a line drawn along the proximal segment of the VA, starting from the internal aperture, and a second line along the descending distal segment through the opercular opening. The midpoint width of the VA was also measured after reformatting the native images along Pöschl’s plane (Fig. 1).

To evaluate intra-observer reproducibility, both observers independently repeated all measurements in a subset of 20 consecutive patients (40 temporal bones) following a 2-month washout period to minimize recall bias.

Intra- and inter-reader agreement were quantitatively assessed using the intraclass correlation coefficient (ICC; two-way random effects, absolute agreement, single measures). Additionally, Bland–Altman plots were constructed to graphically depict the distribution and show the mean and 95% limits of agreement of measurement differences between observers.

## Results

The primary outcome measure of this study was the ATVA. For Reader 1, the mean ATVA was 104.7° for both the right and left ears. For Reader 2, the mean ATVA was 107.7° for the right ear and 110.6° for the left ear. The overall mean ATVA, averaged across both readers and both ears, was 106.9° (Table 1). Reader 2 systematically recorded slightly higher ATVA values compared to Reader 1, although the overall distribution and variability of measurements were comparable between the two readers.

**Table 1:** Descriptive statistics of all VA measurements

Measurements	Reader 1 right (mean ± sd)	Reader 1 left (mean ± sd)	Reader 2 right (mean ± sd)	Reader 2 left (mean ± sd)	Ear-average (mean ± sd)
ATVA (°)	104.7 ± 11.3	104.7 ± 10.8	107.7 ± 12.3	110.6 ± 12.3	106.9 ± 11.7
Internal aperture width (mm)	0.35 ± 0.07	0.37 ± 0.08	0.45 ± 0.19	0.45 ± 0.20	0.41 ± 0.14
Axial midpoint width (mm)	0.69 ± 0.32	0.58 ± 0.27	0.63 ± 0.28	0.58 ± 0.23	0.63 ± 0.28
Pöschl-plane midpoint width (mm)	0.66 ± 0.24	0.63 ± 0.23	0.54 ± 0.21	0.53 ± 0.17	0.59 ± 0.21
Opercular width (mm)	1.2 ± 0.48	1.1 ± 0.39	1.6 ± 0.82	1.4 ± 0.71	1.32 ± 0.60

Secondary outcomes included additional morphometric parameters of the vestibular aqueduct: internal aperture width, axial midpoint width, Pöschl-plane midpoint width, and opercular width. The overall mean values for these parameters were 0.41 ± 0.14 mm for the internal aperture width, 0.63 ± 0.28 mm for the axial midpoint width, 0.59 ± 0.21 mm for the Pöschl-plane midpoint width, and 1.32 ± 0.60 mm for the opercular width. While the midpoint measurements demonstrated relatively consistent distributions between readers, Reader 2 consistently reported marginally larger values for several parameters (Table 1).

**Fig. 2:**
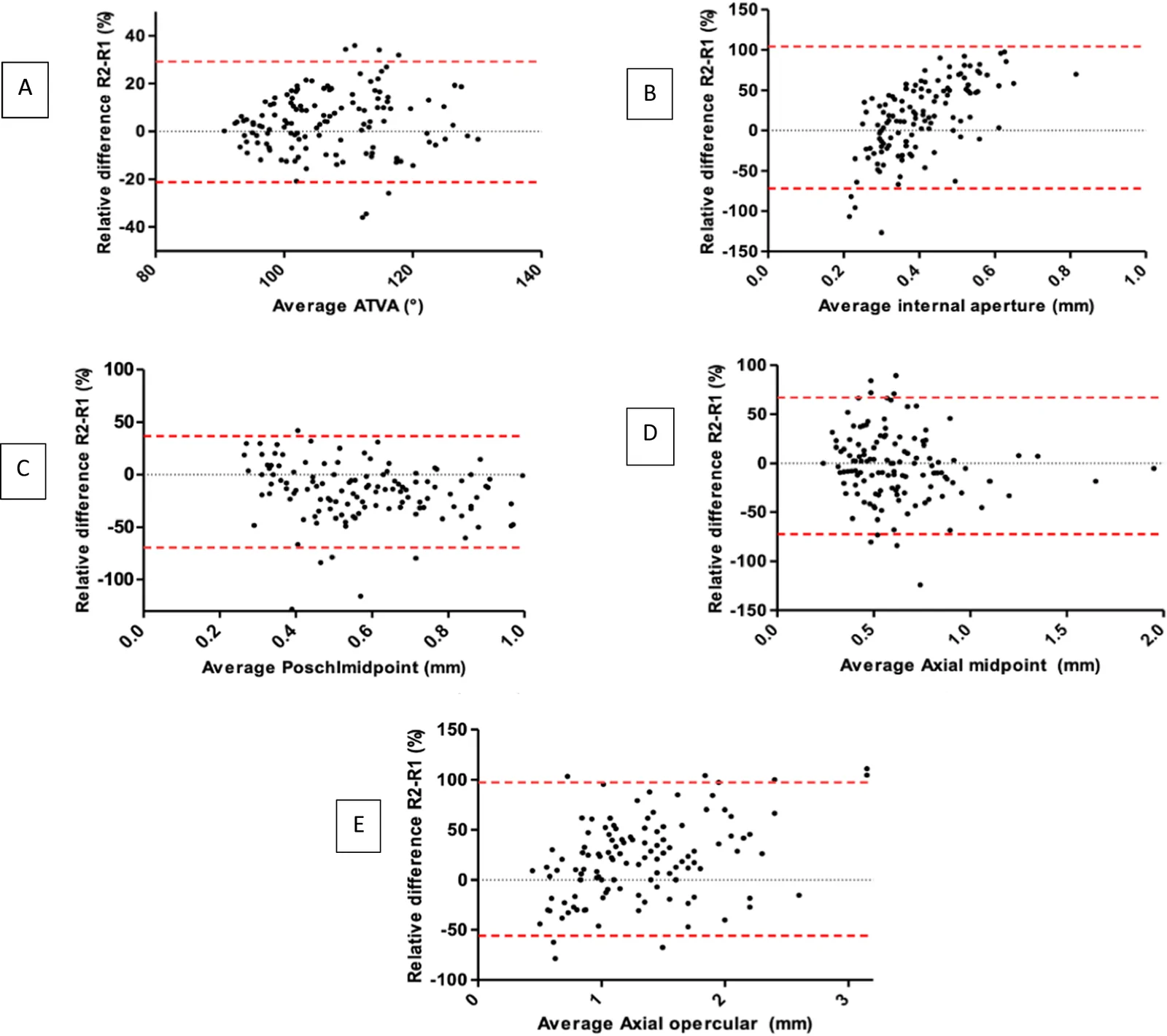
Bland–Altman plots. **A** ATVA (°), **B** internal aperture (mm), **C** Pöschl midpoint (mm), **D** axial midpoint (mm), **E** axial opercular (mm)

When analyzed per ear, the ICC for the ATVA was 0.12 for the right ear and 0.36 for the left ear, indicating poor interobserver agreement (Table 2). Combined analysis of both ears yielded an overall ICC of 0.24, further confirming low interobserver reliability for ATVA measurements. Bland–Altman analysis (Fig. 2) revealed a systematic bias, with Reader 2 consistently recording larger ATVA values than Reader 1. The mean interobserver difference was approximately 4.3°, with wide limits of agreement, reflecting substantial variability in individual measurements.

**Table 2:** Interobserver agreement for VA measurements

Measurement	ICC right ear	ICC left ear	Interpretation
ATVA (°)	0.12	0.36	Poor
Internal aperture width (mm)	0.05	0.13	Poor
Axial midpoint width (mm)	0.83	0.47	Good (right)/moderate to poor (left)
Pöschl-plane midpoint width (mm)	0.65	0.68	Moderate
Axial opercular width (mm)	0.37	0.32	Poor

The axial VA midpoint width demonstrated good agreement for the right ear (ICC = 0.83) and moderate to poor agreement for the left ear (ICC = 0.47). The Pöschl-plane midpoint width showed good agreement, with ICC values of 0.65 and 0.68 for the right and left ears, respectively. Bland–Altman analysis for these midpoint measurements indicated relatively small mean differences between readers and narrower limits of agreement, suggesting greater measurement consistency compared to other parameters.

In contrast, the internal aperture width exhibited poor interobserver agreement, with ICC values of 0.05 for the right ear and 0.13 for the left ear. Similarly, the opercular width demonstrated low reproducibility, with ICC values of 0.37 and 0.32 for the right and left ears, respectively. Bland–Altman analyses for these parameters revealed wide limits of agreement and substantial interobserver variability.

Overall, the ICC and Bland–Altman analyses indicate that midpoint measurements obtained in the axial and Pöschl planes exhibited higher interobserver reproducibility compared to measurements of the internal aperture and opercular width.

For Reader 1, intra-observer agreement for the ATVA was good, with an ICC of 0.80. Among the secondary morphometric parameters, the Pöschl-plane midpoint width also demonstrated good reproducibility (ICC = 0.73). In contrast, moderate agreement was observed for the axial midpoint width (ICC = 0.55), the axial opercular width (ICC = 0.61), and the internal aperture width (ICC = 0.46) (Table 3).

**Table 3:** Intra-observer agreement for VA measurements

Measurement	ICC Reader 1	ICC Reader 2	Interpretation
ATVA (°)	0.80	0.27	Good (R1)/poor (R2)
Internal aperture width (mm)	0.46	0.55	Moderate
Axial midpoint width (mm)	0.55	0.58	Moderate
Pöschl-plane midpoint width (mm)	0.73	0.80	Good
Axial opercular width (mm)	0.61	0.53	Moderate

For Reader 2, intra-observer agreement for the ATVA was poor (ICC = 0.27). However, measurements of VA diameters demonstrated moderate to good reproducibility, with ICC values of 0.55 for the internal aperture width, 0.58 for the axial midpoint width, 0.80 for the Pöschl-plane midpoint width, and 0.53 for the axial opercular width (Table 3).

Overall, the Pöschl-plane midpoint measurement exhibited the highest intra-observer reproducibility for both readers. In contrast, ATVA measurements displayed greater variability, particularly for Reader 2.

## Discussion

PCCT has demonstrated superior visualization of normal temporal bone structures compared to EICT, because of its higher inherent spatial resolution [8]. This enhanced resolution suggests that PCCT may enable a more comprehensive assessment of the VA course.

In the present study, measurements were performed in the plane parallel to the lateral semicircular canal, consistent with methodologies employed in previous investigations [1, 2, 4, 6, 7]. The mean ATVA measurements obtained align with values previously reported in normal subjects and control patients (Table 4).

**Table 4:** Reported ATVA values, compared to the results in this study

Bächinger et al (2019)	102.3 ± 9.8
Jung et al (2023)	108 ± 15.1
Xia et al (2024)	111 ± 18.6
Hung et al (2024)	119.31 ± 15.1
Juliano et al (2026)	98.2 ± 6
This study	106.9 ± 11.7

**Fig. 3:**
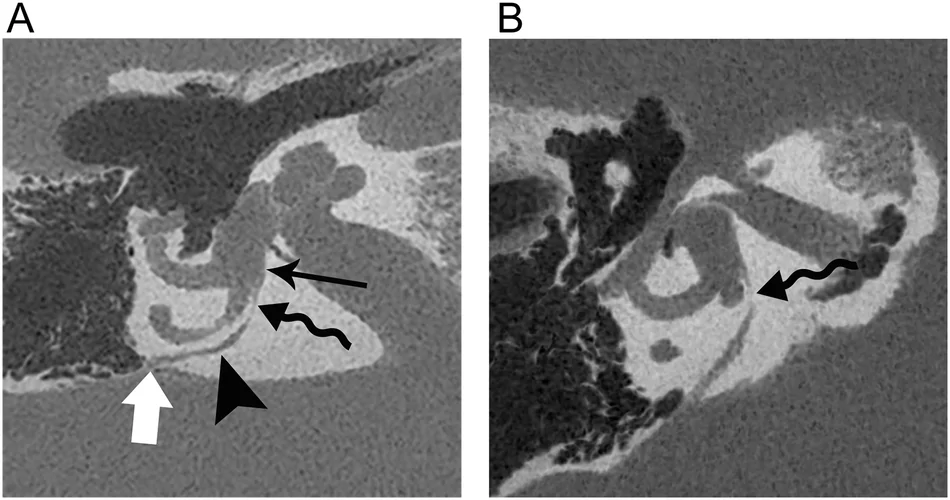
MINIPs showing the course of VA. In patient **A**, the entire course is visible. Internal aperture (black arrow), proximal segment (curved arrow), distal segment (arrowhead), operculum (white arrow). In patient **B**, part of the proximal segment (curved arrow) is not well visible. Note the gradual transition between the proximal and distal segments

Interobserver reproducibility for ATVA measurements was found to be suboptimal, with one observer demonstrating poor intra-observer agreement. Several factors may contribute to this limited reproducibility. In the plane aligned with the lateral semicircular canal, the oblique course of the VA precludes its visualization within a single image, necessitating scrolling through sequential slices to optimally position measurement lines along both the proximal and distal segments. Also, in some patients, parts of the proximal VA segment may still be below the spatial resolution threshold of PCCT, complicating the precise determination of a line accurately reflecting the course of this segment. Furthermore, the junction between the proximal and distal segments of the VA forms a gradual curve, potentially hampering the determination of the angle between these segments. Figure 3 shows minimal intensity projections (MINIP) derived from native PCCT images, calculated in two patients, depicting the VA trajectory in a single plane and illustrating these points. As methodological consistency with previous studies was considered essential, MINIP’s were not used for data generation in this study.

In addition, the broader distal segment of the VA does not always follow a straight trajectory, requiring observers to subjectively place a line best approximating its course, possibly further contributing to measurement inconsistency.

Limited information is available in the literature regarding the interobserver reproducibility of ATVA measurements. Juliano et al [1] demonstrated excellent inter-reader agreement (ICC = 0.92) by standardizing the trajectory of the proximal segment. Specifically, the authors utilized a consistent angular approach, positioning a line at 14.3° relative to the medial border of the vestibule across all patient assessments. This methodological consistency likely minimized variability, as it may not fully account for anatomical diversity in proximal segment morphology. Jung et al [6] reported moderate interrater reliability (ICC = 0.54). Their approach relied on predefined geometric shapes within the ATVA measurement software, with observed variability attributed to subjective manual fitting of these shapes to computed tomography (CT) images. This finding underscores the potential influence of user-dependent factors on measurement consistency. De Pont et al [2] reported on intra-observer reproducibility, achieving a substantial agreement (Cohen’s kappa (κ) = 0.88). Their protocol involved reassessing 20% of cases and categorizing results into three angular groups: < 120°, 120–140°, and > 140°. Their approach similarly constrained anatomical variability by treating the proximal segment as a predefined shape.

The use of predefined shapes in these studies mitigated one source of measurement variability. However, this standardization may compromise the validity of the results, as it does not account for potential anatomical variations in the proximal segment’s trajectory. Consequently, the reported ATVA values may not fully reflect the true anatomical angle, introducing a trade-off between reproducibility and anatomical fidelity. Further research is warranted to explore alternative methodologies that balance reproducibility with anatomical accuracy.

Potential improvements in reproducibility could be achieved by performing ATVA measurements on individualized image planes. For instance, Huang et al [9] conducted measurements on sagittal oblique images, although the reproducibility of their approach was not reported. Alternatively, the use of MINIPs, as demonstrated in Fig. 3, may maximize visualization of the VA course and reduce measurement variability.

An alternative approach may be measuring the retrolabyrinthine bone thickness as a marker for differentiating the hypoplastic from the degenerative endotype of Menière’s disease. Although this parameter was not assessed in the present study, Juliano et al [10] reported that it is strongly associated with ATVA-based endotyping, with mean values of 0.8 mm in the hypoplastic endotype and 2.0 mm in the degenerative endotype, and with excellent reliability. Measuring the retrolabyrinthine bone thickness is likely to be more reliable than subjective line placement along a small and curved vestibular aqueduct and may therefore represent a more robust and clinically practical marker for MD endotyping.

Measurement of the internal opening of the VA proved to be challenging due to its diminutive size. Minor variations in cursor placement may result in substantial changes in measured values, likely accounting for the observed poor measurement reproducibility. Also, the mean value obtained in this study (mean: 0.4 mm) was slightly higher than those reported in the literature (0.1–0.2 mm [11]; 0.3 mm [12]).

Poor reproducibility of the opercular segment measurements may be attributed to considerable anatomical variability in this region. Previous studies have demonstrated significant differences in measured sizes depending on the imaging plane used [12].

Moderate to good reproducibility was observed for the axial midpoint width and the midpoint width in the Pöschl plane. These measurements are routinely employed in clinical practice to assess potential VA enlargement in patients with sensorineural hearing loss. The values obtained in this study are consistent with those reported in earlier investigations [13–15].

In conclusion, estimation of the ATVA using PCCT images yielded results comparable to those of previous studies, suggesting that this technique may be suitable for broadly categorizing patients with Menière’s disease into hypoplastic or degenerative endotypes. However, further research is warranted to develop a more reproducible method for ATVA measurement on PCCT images.

## Data Availability

All data generated in this study are reported in this manuscript.

## Abbreviations

ATVA: Angular trajectory of the vestibular aqueduct
EICT: Energy-integrating computed tomography
ICC: Intraclass correlation coefficient
MD: Menière’s disease
MD-dp: Menière’s disease degenerative endotype
MD-hp: Menière’s disease hypoplastic endotype
MINIP: Minimum intensity projection
PCCT: Photon-counting computed tomography
VA: Vestibular aqueduct
